# Association between genetic risk and adherence to the Dietary Approaches to Stop Hypertension diet for developing venous thromboembolism

**DOI:** 10.1016/j.rpth.2025.102731

**Published:** 2025-03-12

**Authors:** Si Li, Minghui Jiang, Yunlong Guan, Xi Cao, Zhonghe Shao, Jun Deng, Xingjie Hao

**Affiliations:** 1 Department of Epidemiology and Biostatistics, School of Public Health, Tongji Medical College, Huazhong University of Science and Technology, Wuhan, Hubei, China; 2 Institute of Hematology, Union Hospital, Tongii Medical College, Huazhong University of Science and Technology, Wuhan, Hubei, China

**Keywords:** additive interaction, gene-diet interaction, genetic susceptibility, UK Biobank, venous thromboembolism

## Abstract

**Background:**

The relationship between diet and venous thromboembolism (VTE) remains unclear, and the joint effects of diet patterns and genetic susceptibility on VTE risk are unknown.

**Objectives:**

Investigate the independent and joint effects of Dietary Approaches to Stop Hypertension (DASH) diet adherence and polygenic risk scores (PRS) on VTE risk.

**Methods:**

A total of 411,539 UK Biobank participants were included. DASH scores were calculated using Food Frequency Questionnaires, and PRS quantified genetic risk. Cox proportional hazard models estimated hazard ratios (HRs) for VTE, assessing interactions between the DASH diet and genetic susceptibility.

**Results:**

During a median follow-up of 13.4 years, 10,543 participants were diagnosed with VTE. Higher DASH scores were associated with a lower VTE risk (HR, 0.87; 95% CI, 0.82-0.92). A low-adherent DASH diet combined with high-genetic risk had the highest VTE risk (HR, 2.78; 95% CI, 2.47-3.14). High DASH scores reduced VTE risk in high-genetic-risk individuals (HR, 0.84; 95% CI, 0.76-0.92). Sex-specific associations were detected in the joint effect and interaction of DASH scores and PRS. Notably, high DASH scores can offset moderate genetic risk among men (HR, 0.79; 95% CI, 0.67-0.94). There were additive interactions between DASH scores and high genetic risk in total subjects and men, while not observed in women.

**Conclusion:**

The DASH diet is associated with reduced VTE risk and can partially offset genetic predisposition. Low adherence to the DASH diet increases VTE risk, particularly in high-genetic-risk individuals. The protective effect of high DASH scores against genetic risks for VTE is more pronounced in males. Precision medicine should consider both diet and genetics for VTE prevention.

## Essentials

• Diet and genetic risk interaction on VTE remains unclear.
• We analyzed 411,539 UK Biobank participants.
• The DASH diet lowered VTE risk despite high genetic risk.
• We found an additive DASH diet–genetic risk interaction in all subjects and men.

## Visual Abstract

**Figure:**
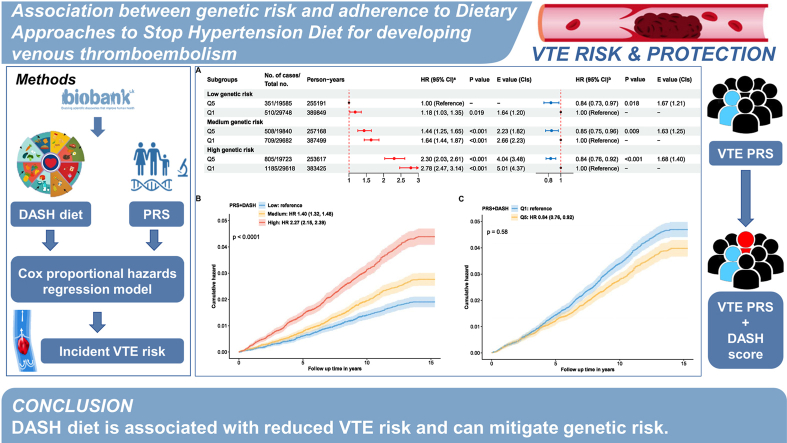

## 1 Introduction

Venous thromboembolism (VTE) is a common medical condition with high morbidity and mortality [1,2], especially among the elderly, with an annual incidence rate comparable to that of stroke of European descent [[3], [4], [5], [6], [7]]. Research on the association between diet and VTE risk has been ongoing and debated [[8], [9], [10], [11], [12]]. Previous studies focusing on single nutrients or specific food components, which only partially represented individual overall diet patterns [[13], [14], [15], [16], [17]], have yielded neutral or inconsistent results [8,12]. Evaluations of dietary patterns by considering multiple food components provide a more comprehensive snapshot of dietary exposures. The Dietary Approaches to Stop Hypertension (DASH) diet plan, high in fruits, vegetables, and low-fat dairy products but low in saturated and total fat, reduces blood pressure and lipid levels [[18], [19], [20]], accompanied by lower rates of cardiovascular-related metabolic diseases [21,22]. However, direct evidence on the associations of the DASH diet with VTE risk remains unclear [8]. To date, only 2 prospective studies have systematically examined associations between the DASH diet and VTE risk, with conflicting results [8,23]. Given that, assessing DASH diet adherence as a risk factor for VTE in large population-based cohorts is necessary to gain insight into the impact of food and nutrient combinations on VTE occurrence and to explore dietary pattern methodologies for VTE prevention.

Genetic predisposition contributes to persistent VTE risk throughout the lifetime [24], with genome-wide association study (GWAS) identifying susceptibility loci [25,26]. These loci form polygenic risk scores (PRS) for estimating genetic VTE risk [26,27], but their predictive performance varies based on environmental factors [8,28]. Nongenetic factors, such as dietary patterns, may modify genetic VTE associations, potentially influencing personalized nutritional guidance for VTE prevention. Therefore, it is imperative to explore the joint effect and interaction of genetic risk and dietary patterns on VTE risk, an aspect often overlooked in previous studies.

Initially, we developed a score to gauge adherence to the DASH diet. We then assessed the associations between DASH scores and VTE in this population-based prospective cohort. Subsequently, we utilized a PRS for VTE to quantify the genetic risk of VTE events. Additionally, our study investigated how dietary patterns and genetic susceptibility jointly affect the development of VTE and the extent to which adherence to the DASH diet modified the impact of genetic susceptibility on VTE risk.

## 2 Methods

### 2.1 Data source and participant inclusion

**Figure 1:**
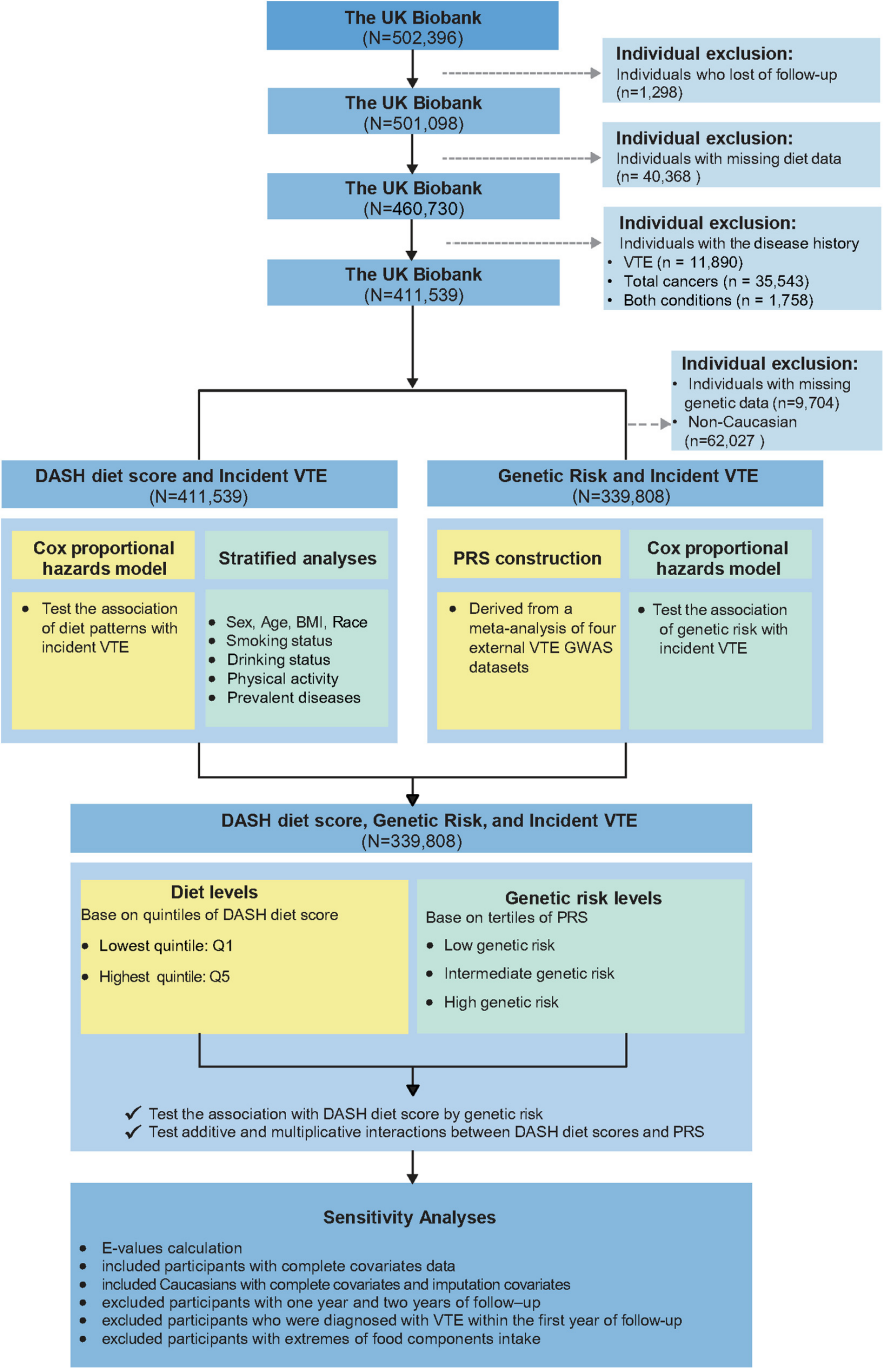
Study population and workflow. BMI, body mass index; DASH, Dietary Approaches to Stop Hypertension; GWAS, genome-wide association study; PRS, polygenic risk scores; Q, quintile; VTE, venous thromboembolism.

The present study was based on data from the UK Biobank, a population-based prospective cohort study of more than half a million community-dwelling participants aged 40 to 69 years recruited between 2006 and 2010 from across the United Kingdom. More details about the technical aspects have been previously described, and participants provided written consent for privacy and confidentiality [29]. The study population and analytical workflow are outlined in Figure 1. We excluded participants who withdrew and those with missing dietary information to maintain data integrity. Additionally, individuals with a history of cancer or previous VTE were excluded due to changes in health status and likely diet plan. Accordingly, a total of 411,539 participants were eligible for inclusion in the current analysis. When combined with genetic data, we further restricted our analysis to Caucasian, who provided complete genetic information to eliminate spurious associations due to differences in genetic architecture across ethnicities. The UK Biobank sample classification for Caucasian (field ID in the UK Biobank: 22006) stems from both principal component analysis and self-declared ethnicity [30]. Participants were followed up from recruitment until the diagnosis of VTE or death or the end of July 9, 2022. Person-time in years from baseline to the first occurrence of VTE diagnosis, death, loss to follow-up, or end of follow-up was calculated for each participant.

### 2.2 Dietary assessment of DASH scores

We utilized reproducible Food Frequency Questionnaire raw data to calculate the DASH dietary concordance score, as per the method outlined by Fung et al. [18,31,32]. The scoring system focused on 7 components in alignment with the DASH diet: high intake of fruits, vegetables, low-fat dairy products, and whole grains, and low intake of red/processed meats, sugar, and sodium [33]. Participants were segmented into quintiles (Qs) based on their intake of each component, with sodium, sugar–sweetened beverages, and red/processed meat intake reverse scored such that higher intake received a lower score. Excluding samples with incomplete dietary information, we derived overall DASH scores by summing up the Q rankings of the different food items. Supplementary Table S1 depicts the scoring methodology and criteria. The personalized DASH score ranges from 7 (low adherence) to 35 (high adherence), defaulting to the Q score when not explicitly specified.

### 2.3 Calculation of PRS

The PRS offers an individual assessment of the genetic predisposition to disease by aggregating genetic risk variants across the genome [34,35]. The standard PRS set for VTE has been calculated for each individual and was publicly available with field ID 26289 in the UK Biobank. To examine the association between genetic risk and disease risk across various levels of genetic susceptibility, the genetic risk was categorized into low (bottom third of the resulting PRS), intermediate (middle third), and high (top third).

### 2.4 VTE definition

We ascertained the initial VTE according to the first occurrence of any code mapped to the 3-character International Classification of Diseases 10th Revision (ICD-10) (category ID: 1712), which was generated by mapping the ICD-10 code in the death register records, read code information in the primary care data, International Classification of Diseases 9th Revision (ICD-9) and ICD-10 codes in the hospital inpatient records (eg, diagnosis or operation code), and self-reported medical conditions reported at the baseline or subsequent visit to the UK Biobank assessment center. VTE cases were defined as having at least 1 of the following ICD-10 diagnosis code: (1) “I26” pulmonary embolism (PE; field ID: 131309), (2) “I80” thrombosis, phlebitis, and thrombophlebitis (field ID: 131397), and (3) “I82” other VTE and thrombosis (field ID: 131401) [36]. The remaining individuals were included in the control group. Finally, our analysis retained 411,539 individuals comprising 400,996 controls and 10,543 VTE cases.

### 2.5 Statistical analysis

Baseline characteristics of the participants were described according to VTE status using the chi-square test or Mann–Whitney U-test, while for the DASH scores, we used the chi-square test or the Kruskal–Wallis test. We reported the count and percentage for categorical variables and the median and IQR for continuous variables.

To examine the association of DASH scores and PRS with incident VTE, we applied the Cox proportional hazards model and calculated hazard ratios (HRs) and 95% CIs. Schoenfeld’s residuals were used to ensure that our model met the proportional hazards assumption. Adherence to the DASH diet was modeled as Qs of the DASH scores in males and females combined, as the findings were similar in analyses when DASH scores were sex-specific (*P* for interaction = .33). For DASH scores, we conducted multivariate Cox proportional hazards regression models, adjusting sequentially for socio-demographic, lifestyle, and health-related risk factors. More details of covariates are described in the Supplementary Methods. Model 1 was crudely adjusted for age and sex. Model 2 was further adjusted for body mass index, ethnicity, Townsend Deprivation Index, smoking status, drinking status, and physical activity. Model 3 was additionally adjusted for the following putative risk factors for VTE: aspirin medication [[37], [38], [39]], systolic blood pressure [[40], [41], [42]], low-density lipoprotein [25,43], and a history of cardiovascular disease (CVD)/diabetes/hypertension/lipidemia [9]. In the PRS combined model, we also adjusted for the genotyping batch and the first 5 genetic principal components. Besides, exposure-response relationships between diet and PRS with the outcome variable were respectively evaluated with a restricted cubic spline to investigate linear or nonlinear associations in the fully adjusted Cox models [44]. The E-value was calculated using the R package “E-Value” [45]. It assesses how strongly an unmeasured confounder must be related to the exposure and outcome to explain an effect estimate. The E-value represents the minimum strength of an association that an unmeasured confounder would need to have with both the exposure and outcome to fully explain the observed association. The formula for the E-value is given by:

E−value=HR+sqrt(HR×[HR−1])

where HR is the hazard ratio. A higher E-value indicates greater confidence in the robustness of the findings, while a lower E-value suggests that a weaker confounder could potentially negate the observed effect. Akaike’s information criterion was used to determine the number of knots for the best model fit, and the median values were used as the reference [46]. To explore potential sources of variation and enhance the robustness of the DASH scores, we conducted several subgroup and sensitivity analyses that are summarized in the Supplementary Methods.

To investigate whether the associations of dietary patterns with VTE might be different according to genetic risk, we cross-classified participants by categories of DASH scores (Q1 and Q5) and PRS (low, medium, and high) and conducted joint analyses. The multiplicative interaction was calculated by modeling a multiplicative term between the DASH scores and PRS categories in the full-adjusted model, and the additive interaction was multiplied by using relative excess risk (RERI), the attributable proportion due to the interaction (AP), and the synergy index [47]. If there was no additive interaction, the CIs of the RERI and AP would include 0, and the CIs of the synergy index would include 1 [48]. To determine the proportion of cases prevented by eliminating the specific risk factors in the population, we calculated the confounders’ adjusted attributable fraction (AAF) using a doubly robust estimator of the attributable fraction function [49]. Details of the calculation and function were reported elsewhere [49].

All the analyses were performed using R Statistical Software (v4.1.2: R Core Team 2021). Statistical significance was defined as a 2-tailed *P* value less than .05.

## 3 Results

### 3.1 Baseline characteristics of participants

Baseline characteristics were shown for 411,539 participants stratified by Qs of the DASH scores in the UK Biobank (Table 1). Participants with higher DASH scores were more likely to be older (mean age, 60 years; 53.4% more than 60 years), females (56.2%), more physically active (40.4%), never-smokers (56.0%), and moderate drinkers (61.5%), with a high proportion of obesity (25.2%) at baseline. We further compared the baseline characteristics of subjects according to VTE status Supplementary Table S2 During a median follow-up of 13.4 years, a total of 10,543 incident VTE cases were recorded. The incident VTE cases were older (mean age, 57.0 years) and had a greater proportion of males (52.2%), smokers (12.3%), and previous drinkers (90.8%), as well as higher rates of obesity (34.7%) and previous aspirin medication (18.2%) at baseline, and more participants who had CVD (9.4%), diabetes (4.3%), hypertension (34.5%), and lipidemia history (19.1%) than participants without VTE. Additionally, they exhibited less physical activity and had a higher Townsend Deprivation Index.

**Table 1:** Comparisons of baseline characteristics according to quintiles of the Dietary Approaches to Stop Hypertension diet scores.

Characteristics, *N* (%) or median (IQR)	Q1 (*N* = 102,134)	Q2 (*N* = 76,014)	Q3 (*N* = 81,949)	Q4 (*N* = 81,658)	Q5 (*N* = 69,784)
Age (y), continuous	54.0 (47.0, 61.0)	56.0 (49.0, 62.0)	57.0 (50.0, 63.0)	58.0 (51.0, 63.0)	60.0 (53.0, 64.0)
Age (y), categories					
<60	69,305 (67.9)	47,607 (62.6)	48,234 (58.9)	44,471 (54.5)	32,540 (46.6)
≥60	32,829 (32.1)	28,407 (37.4)	33,715 (41.1)	37,187 (45.5)	37,244 (53.4)
Sex					
Female	59,198 (58.0)	41,943 (55.2)	44,446 (54.2)	36,050 (44.1)	39,220 (56.2)
Male	42,936 (42.0)	34,071 (44.8)	37,503 (45.8)	45,608 (55.9)	30,564 (43.8)
Ethnicity					
Other	20,798 (20.4)	13,083 (17.2)	13,297 (16.2)	12,677 (15.5)	10,610 (15.2)
CaucasianCaucasian	81,336 (79.6)	62,931 (82.8)	68,652 (83.8)	68,981 (84.5)	59,174 (84.8)
BMI (kg/m^2^)					
18.5-24.9	33,507 (32.8)	25,225 (33.2)	27,601 (33.7)	26,525 (32.5)	22,396 (32.1)
<18.5	619 (0.6)	392 (0.5)	412 (0.5)	330 (0.4)	300 (0.4)
25-29.9	42,976 (42.1)	32,468 (42.7)	34,764 (42.4)	35,570 (43.6)	29,256 (41.9)
≥30	24,443 (23.9)	17,587 (23.1)	18,819 (23.0)	18,886 (23.1)	17,562 (25.2)
Missing	589 (0.6)	342 (0.4)	353 (0.4)	347 (0.4)	270 (0.4)
Smoking status					
Never	53,530 (52.4)	42,368 (55.7)	46,463 (56.7)	45,674 (55.9)	39,058 (56.0)
Previous	32,370 (31.7)	25,380 (33.4)	28,023 (34.2)	29,210 (35.8)	25,969 (37.2)
Current	15,932 (15.6)	8071 (10.6)	7264 (8.9)	6540 (8.0)	4536 (6.5)
Missing	302 (0.3)	195 (0.3)	199 (0.2)	234 (0.3)	221 (0.3)
Drinking status					
Nondrinkers	21,748 (21.3)	13,764 (18.1)	14,063 (17.2)	13,014 (15.9)	12,240 (17.5)
Moderate	59,312 (58.1)	46,604 (61.3)	51,011 (62.2)	50,851 (62.3)	42,942 (61.5)
Heavy	21,002 (20.6)	15,614 (20.5)	16,838 (20.5)	17,778 (21.8)	14,574 (20.9)
Missing	72 (0.1)	32 (0.0)	37 (0.0)	15 (0.0)	28 (0.0)
Physical activity					
Low	20,394 (20.0)	12,545 (16.5)	12,277 (15.0)	10,737 (13.1)	7779 (11.1)
Moderate	34,234 (33.5)	26,441 (34.8)	28,375 (34.6)	27,937 (34.2)	22,637 (32.4)
High	28,240 (27.6)	23,767 (31.3)	27,669 (33.8)	30,468 (37.3)	28,169 (40.4)
Missing	19,266 (18.9)	13,261 (17.4)	13,628 (16.6)	12,516 (15.3)	11,199 (16.0)
Townsend index	-1.8 (-3.5, 1.1)	-2.2 (-3.7, 0.4)	–2.3 (-3.7, 0.1)	-2.4 (-3.8, -0.0)	-2.4 (-3.8, -0.1)
SBP (mm Hg)	135.5 (123.5, 146.0)	136.0 (124.5, 147.0)	136.0 (125.0, 148.0)	136.0 (126.5, 149.5)	137.0 (127.0, 150.5)
DBP (mm Hg)	82.0 (75.5, 88.0)	82.0 (75.5, 88.5)	82.0 (75.5, 88.5)	82.0 (76.0, 88.5)	82.0 (75.5, 88.5)
LDL (mmol/L)	3.5 (3.0, 4.1)	3.5 (3.0, 4.1)	3.5 (3.0, 4.1)	3.5 (3.0, 4.0)	3.5 (2.9, 4.0)
Aspirin					
Never	89,439 (87.6)	66,059 (86.9)	70,597 (86.1)	68,807 (84.3)	57,725 (82.7)
Previous	11,588 (11.3)	9243 (12.2)	10,630 (13.0)	12,093 (14.8)	11,461 (16.4)
Missing	1107 (1.1)	712 (0.9)	722 (0.9)	758 (0.9)	598 (0.9)
History of CVD					
No	96,493 (94.5)	71,755 (94.4)	77,219 (94.2)	76,181 (93.3)	64,931 (93.0)
Yes	5,641 (5.5)	4,259 (5.6)	4,730 (5.8)	5,477 (6.7)	4,853 (7.0)
History of diabetes					
No	100,454 (98.4)	74,591 (98.1)	80,191 (97.9)	79,178 (97.0)	66,311 (95.0)
Yes	1,680 (1.6)	1,423 (1.9)	1,758 (2.1)	2,480 (3.0)	3,473 (5.0)
History of hypertension					
No	79,102 (77.4)	57,714 (75.9)	61,342 (74.9)	59,151 (72.4)	48,420 (69.4)
Yes	23,032 (22.6)	18,300 (24.1)	20,607 (25.1)	22,507 (27.6)	21,364 (30.6)
History of lipidemia					
No	89,339 (87.5)	65,925 (86.7)	70,530 (86.1)	68,786 (84.2)	57,983 (83.1)
Yes	12,795 (12.5)	10,089 (13.3)	11,419 (13.9)	12,872 (15.8)	11,801 (16.9)

BMI, body mass index; CVD, cardiovascular disease; DBP, diastolic blood pressure; LDL, low-density lipoprotein; Q, quintile; SBP, systolic blood pressure; IQR, inter-quartile range.

### 3.2 Association of DASH scores with incident VTE

**Figure 2:**
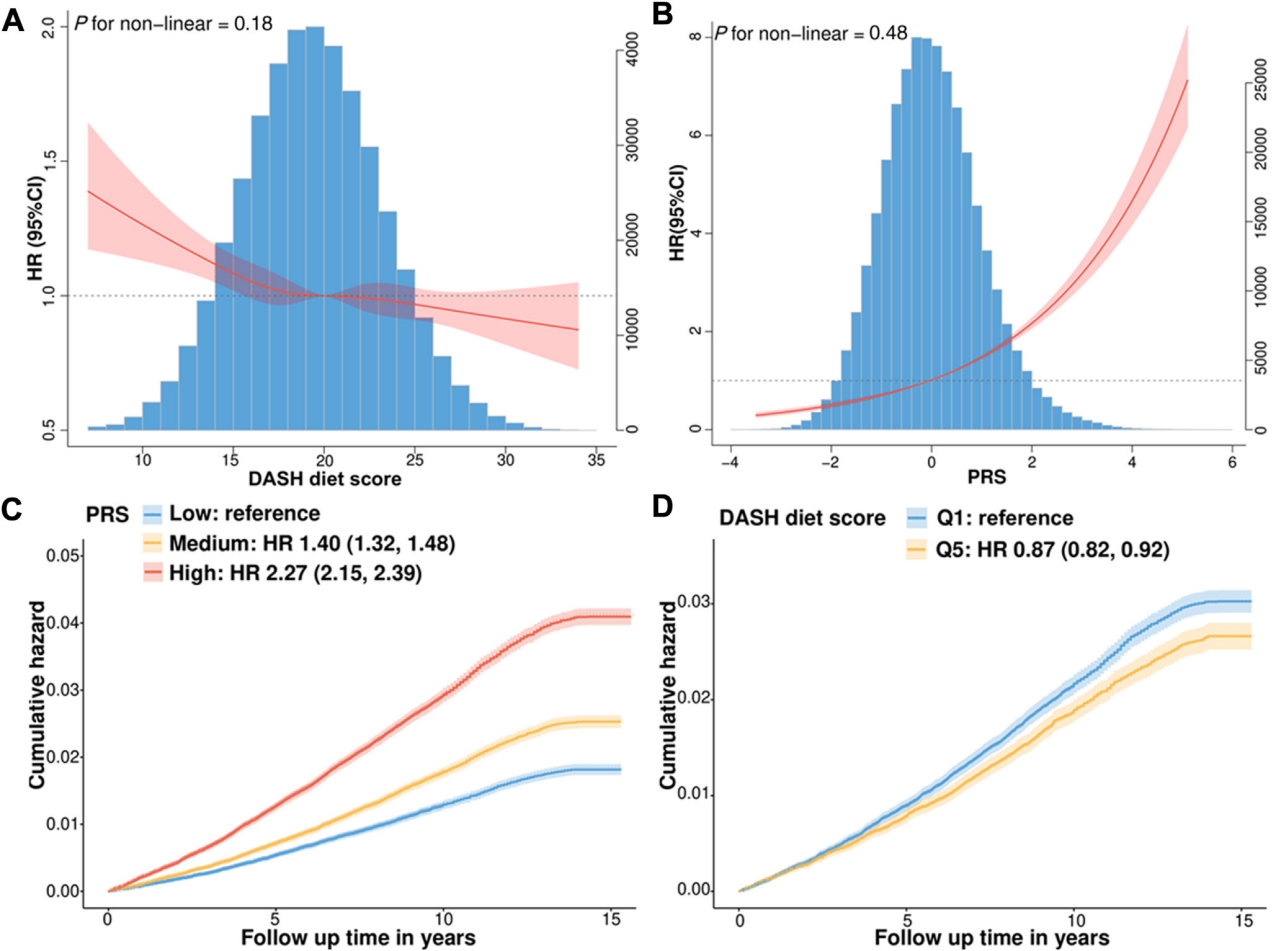
Effect of Dietary Approaches to Stop Hypertension (DASH) scores and polygenic risk scores (PRS) on the risk of venous thromboembolism (VTE) incidence. (A) The exposure-response relationship of DASH scores with the risk of incident VTE. (B) The exposure-response relationship of PRS with the risk of incident VTE. The blue histogram represents the distribution of the exposures. Solid red lines are hazard ratios (HRs) assessed using a restricted cubic spline analysis. (C) Cumulative VTE hazard for quintile (Q) 1 and Q5 of the DASH scores. (D) Cumulative VTE hazard for high, intermediate, and low genetic risk groups. All HRs and 95% CIs were estimated using Cox proportional hazard models adjusted for age, sex, ethnicity, body mass index, Townsend Deprivation Index, smoking status, drinking status, physical activity, aspirin medication, systolic blood pressure, low-density lipoprotein, ethnicity, and history of cardiovascular disease, diabetes, hypertension, or lipidemia (HRs for PRS were further adjusted by genotyping batch and the first 5 principal components of ancestry).

We first investigated the associations of DASH scores with the risk of VTE events. After adjustment for potential confounders, a significant inverse association was observed between DASH scores and the risk of VTE in a linear manner, with an HR of 0.87 (95% CI, 0.82-0.92) when comparing the highest with lowest Qs (Table 2 and Figure 2A). To further validate the association over follow-up time, we conducted cumulative risk analyses, which showed that the risk of VTE over time was lower for participants with Q5 DASH scores than those with Q1 DASH scores (Figure 2C). Overall, these results demonstrated that a high-adherent DASH diet protects against VTE incidence over 15 follow-up years.

**Table 2:** Associations of Dietary Approaches to Stop Hypertension scores and polygenic risk scores with risk of incident venous thromboembolism among the total participants.

Exposure	No. of cases/total no.	Person-year	Incidence rate % (95% CI)	Model 1 HR (95% CI)^c^	Model 2 HR (95% CI)^c^	Model 3 HR (95% CI)^c^	E-value (CI)^d^
DASH scores^a^							
continuous/per 1 SD	10,543/411,539	5,354,025	1.97 (1.93, 2.01)	**0.90 (0.87, 0.93)**	**0.92 (0.89, 0.95)**	**0.92 (0.89, 0.95)**	1.40 (1.29)
Q1	2,588/102,134	1,328,822	1.95 (1.87, 2.02)	1.00 (ref.)	1.00 (ref.)	1.00 (ref.)	
Q2	1,899/76,014	990,746	1.92 (1.83, 2.00)	**0.92 (0.87, 0.98)**	**0.95 (0.90, 1.01)**	**0.95 (0.90, 1.01)**	1.28 (1.00)
Q3	2,042/81,949	1,067,936	1.91 (1.83, 2.00)	**0.88 (0.83, 0.94)**	**0.92 (0.87, 0.97)**	**0.92 (0.87, 0.97)**	1.40 (1.19)
Q4	2,109/81,658	1,061,360	1.99 (1.90, 2.07)	**0.87 (0.82, 0.92)**	**0.90 (0.85, 0.96)**	**0.90 (0.85, 0.96)**	1.45 (1.25)
Q5	1,905/69,784	905,162	2.11 (2.01, 2.20)	**0.86 (0.81, 0.91)**	**0.87 (0.82, 0.92)**	**0.87 (0.82, 0.92)**	1.57 (1.39)
PRS^b^							
continuous/per 1 SD	8,970/339,808	4,426,422	2.03 (1.98, 2.07)	**1.46 (1.43, 1.48)**	**1.45 (1.42, 1.48)**	**1.45 (1.42, 1.48)**	2.26 (2.20)
Low	1,924/113,156	1,480,819	1.30 (1.24, 1.36)	1.00 (ref.)	1.00 (ref.)	1.00 (ref.)	
Medium	2,710/113,492	1,480,571	1.83 (1.76, 1.90)	**1.41 (1.33, 1.49)**	**1.40 (1.32, 1.48)**	**1.40 (1.32, 1.48)**	2.14 (1.97)
High	4,336/113,160	1,465,032	2.96 (2.87, 3.05)	**2.29 (2.17, 2.42)**	**2.27 (2.15, 2.39)**	**2.27 (2.15, 2.39)**	3.96 (3.72)

DASH, Dietary Approaches to Stop Hypertension; HR, hazard ratio; PRS, polygenic risk scores; Q, quintile; Ref, reference; VTE, venous thromboembolism.

a SD and quintiles of DASH scores: SD_DASH_ = 3.842, Q1 (≤17); Q2 (18∼19); Q3 (20∼21); Q4 (22∼23); Q5 (≥24).

b SD and tertiles of PRS: SD_PRS_ = 0.998, high (≥0.384); intermediate (-0.441 to ∼0.384); low (<-0.441).

c Model 1: age and sex; model 2: model 1 + body mass index + smoking + drinking + Townsend Deprivation Index + physical activity + ethnicity; model 3: model 2 + systolic blood pressure + low-density lipoprotein + aspirin medication + coronary artery disease history + diabetes history + hypertension history + lipidemia history. The boldface values denote significant differences (*P* < .05).

d The E-value was calculated based on the full-adjusted HR and lower bound of the 95% CI.

As depicted in Supplementary Figure S1, higher DASH scores lowering the risk of VTE were comparably prominent in older participants, males, Caucasian, nonsmokers, moderate drinkers, no aspirin users, those with previous hypertension, those with nonobesity, more physically active, and those without a history of CVD, diabetes, or lipidemia (*P* for interaction > .05). Despite the slightly attenuated effect in some sensitivity analyses, the associations remained generally consistent with the main analyses (Supplementary Table S6). The E-value implies that a substantial unmeasured confounding effect would be required to nullify the estimated association, measured by the HR, given the effects of the measured confounders [45,50]. Among 411,539 individuals, 10,543 were VTE cases, including 4,869 deep vein thrombosis (DVT) alone, 4,593 PE alone, and 1081 PE with DVT. The high DASH score significantly reduced the 20% PE alone risk (HR, 0.80; 95% CI, 0.73-0.88) but showed no significant effects for DVT alone or PE with DVT. Our finding highlights the potential role of the DASH diet as a primary prevention strategy for PE alone. All multivariable HRs (95% CIs) for VTE were less than their respective E-values (CIs), indicating that the associations observed between DASH score or PRS and VTE, adjusted for measured confounders, are robust against potential unmeasured confounding.

### 3.3 Association of genetic risk with incident VTE

Compared with the low genetic risk group, participants with medium genetic risk (HR, 1.40; 95% CI, 1.32-1.48) or high genetic risk (HR, 2.27; 95% CI, 2.15-2.39) were observed to have a higher risk of incident VTE (Table 2). Likewise, a monotonically linear increase was observed across the PRS on a continuous scale (nonlinear *P* = .48; Figure 2B). Furthermore, the cumulative VTE risk gradually ascended in association with high PRS, suggesting that VTE-PRS could achieve efficient risk stratification for the VTE population (Figure 2D). As shown in Supplementary Table S3, subjects with *FV Leiden* polymorphism were associated with increased VTE risk (HR, 2.40; 95% CI, 1.89-2.19). Significant associations were observed in the additive model for *FV Leiden* and prothrombin *G20210A* heterozygotes, with increased VTE risk (genotype CT vs CC, HR, 2.00; 95% CI, 1.86-2.16; genotype GA vs GG, HR, 1.42; 95% CI, 1.29-1.55). The risk of VTE associated with deficiencies in natural anticoagulants exceeds 6-fold (HR, 7.53; 95% CI, 6.41-8.85).

### 3.4 Joint association and interaction of DASH scores and PRS with VTE

**Figure 3:**
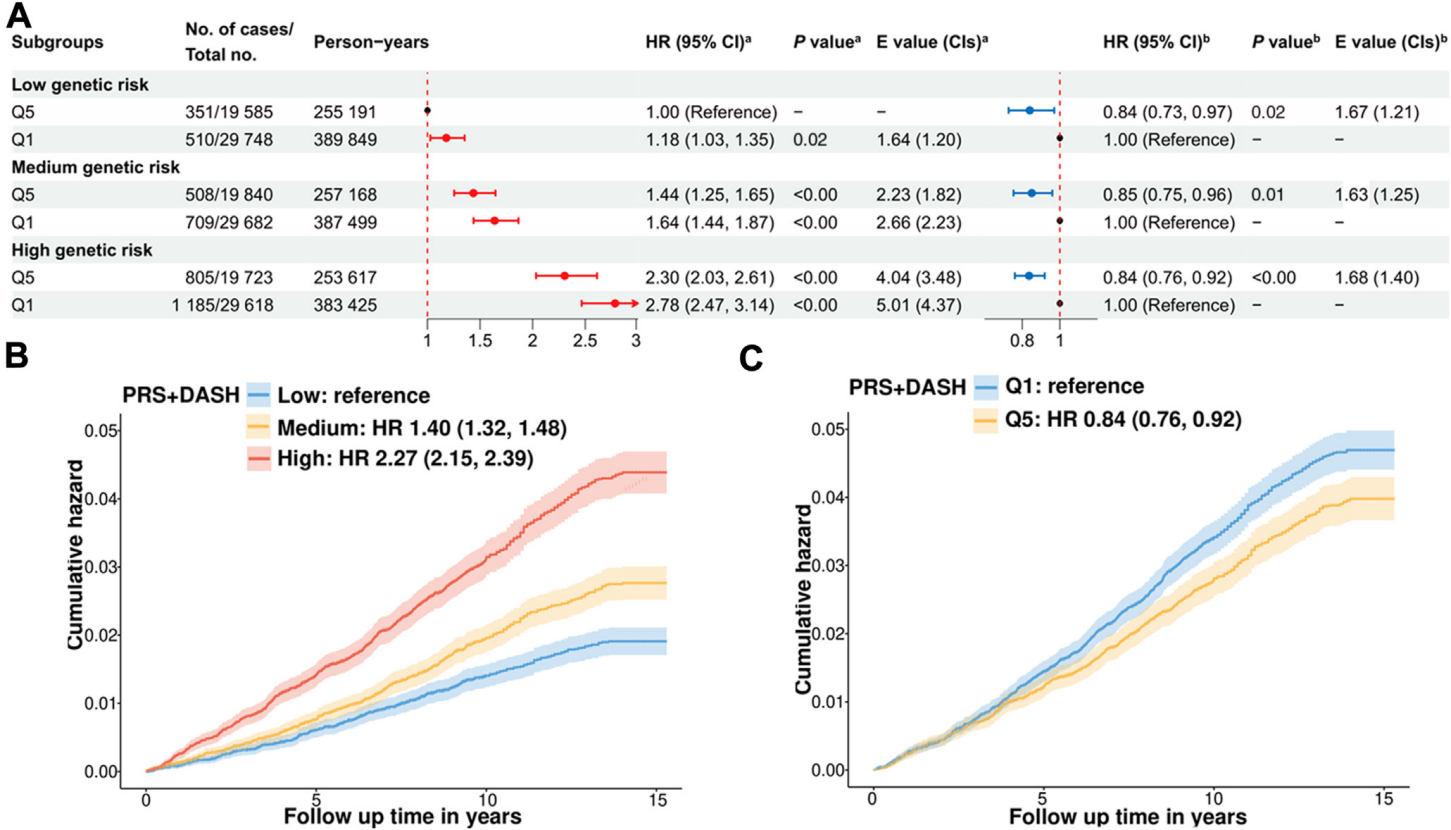
Joint effects and interaction of Dietary Approaches to Stop Hypertension (DASH) scores and genetic risk on venous thromboembolism (VTE) incidence. (A) Joint effect and interaction of DASH scores and genetic risk on VTE incidence. (B) Cumulative VTE hazard for different genetic risks within the highest quintile (Q) of DASH. (C) Cumulative VTE hazard for different DASH scores within the low genetic risk. All hazard ratios (HRs), *P* values, and E-values were adjusted for age, body mass index, sex, ethnicity, the Townsend Deprivation Index, smoking status, drinking use, physical activity, systolic blood pressure, low-density lipoprotein, aspirin medication, a history of cardiovascular disease, diabetes, hypertension or lipidemia, genotyping batch, and the first 5 genetic principal components. ^a^Participants with the highest Q of DASH scores and low genetic risk were set as the reference. ^b^Participants with the lowest Q of DASH scores in each genetic risk stratification were set as the reference. PRS, polygenic risk scores.

The joint effect of genetic factors and DASH scores showed a dose-response association, with higher genetic risk and lower adherence to the DASH diet increasing VTE risk gradually (Figure 3A). The risk of VTE was highest in those with high PRS and low DASH scores (HR, 2.78; 95% CI, 2.47-3.14) compared with those with low PRS and a high-adherent DASH diet. In particular, the protective effects of a high-adherent DASH on VTE were generally consistent across all genetic profiles, suggesting that the risk of VTE associated with genetic predisposition could be mitigated by adopting a DASH diet. Cumulative VTE risk over time confirmed lower VTE risk in participants with high dietary scores and low genetic risk (Figure 3B, C). Similarly, a decreasing trend of VTE risk associated with a low- and high-adherent DASH diet was observed when stratified by categories of PRS (Supplementary Figure S2A), and the HRs of VTE associated with high PRS increased sharply across DASH diet categories (Supplementary Figure S2B). Supplementary Table S4 indicates that high DASH scores did not show a significant protective effect in individuals heterozygous for FV Leiden, prothrombin G20210A, or those with coagulation defects, which only accounted for a small proportion of total events (∼0.2% of the study population).

Some interactions and sex-specific associations were detected between DASH scores and PRS with VTE risk (Supplementary Table S7, Supplementary Figure S3). The additive association is presented in Supplementary Table S7, with high DASH scores and low genetic risk as reference. A positive additive interaction was identified only in those exposed to high genetic risk and low DASH scores, with a 0.30 RERI due to the additive interaction (RERI, 0.29; 95% CI, 0.03-0.55). Moreover, a substantial portion (AP, 0.11; 95% CI, 0.01-0.20) of VTE events was attributable to an additive interaction of poor-adherent DASH diet and high genetic risk. In sex-specific analyses, we found a significant additive interaction between low DASH scores and high PRS in males (RERI, 0.46; 95% CI, 0.08-0.85) but not in females (RERI, 0.11; 95% CI, −0.33 to 0.54). The joint impact of low DASH scores and genetic predisposition was more pronounced in males (Supplementary Figure S3). Notably, high DASH scores significantly offset moderate genetic risk among men, with an HR of 0.79 (95% CI, 0.67-0.94), while high DASH scores did not significantly counterbalance low genetic risk (*P* = .09). As for females, the protective effect of high DASH sores was only significant for high genetic risk (HR, 0.84; 95% CI, 0.76-0.92). These findings suggest that genetic predisposition is not deterministic of VTE incidence, highlighting the potential benefits of a high-adherent DASH diet in offsetting genetic VTE risks, especially for males and the high-genetic-risk group.

### 3.5 AAF for incident VTE

The confounder AAF was estimated using multivariable Cox proportional hazards regression models (Supplementary Figure S4). The AAF for low DASH scores remained consistent over 15 follow-up years, indicating a potential elimination of more than 8% of VTE events by improving a low-adherent DASH diet. Similarly, high genetic risk contributed to more than 38% of VTE events compared with low genetic risk. The stable AAF over time suggests a lasting impact of both the DASH diet and genetic factors on VTE occurrence, underscoring the importance of considering these factors in long-term VTE risk assessment and prevention.

## 4 Discussion

This large-scale, prospective cohort study spanned over 15 years and aimed to investigate how DASH dietary patterns and genetic susceptibility contribute to the risk of VTE independently and jointly. Both DASH scores and PRS showed linear exposure-response associations with incident VTE, with decreasing and increasing trends, respectively. Our joint analyses revealed that the highest risk occurred among individuals with high genetic risk and low DASH diet. A high-adherent DASH diet could offset VTE risk attributed to genetic predisposition in stratified analysis. Additionally, the interactions between PRS and DASH scores in incident VTE were quantitatively evaluated.

Limited observational studies have produced conflicting findings regarding the relationship between dietary factors and VTE events [16,23,51,52]. To date, only 1 prospective evaluation found that high DASH scores were associated with low risks of VTE [8], which is in line with our findings. With a larger sample size and more diverse populations, our study also examined the potential interaction between genetic susceptibility and the DASH diet. Diet might modify the risk of VTE through intake of specific nutrients, altering levels of hemostatic and fibrinolytic biomarkers, or being associated with obesity [53]. The DASH diet significantly overlapped with previously identified dietary risk factors for VTE, such as sodium, red and processed meat, and trans fatty acids [15,23,54], and protective or neutral factors like low-fat dairy intake and fruits and vegetables [12,14,23], which might explain the linear dose-response association between DASH scores and VTE risk observed in our findings and reinforce the potential clinical value of adopting the DASH diet for VTE protection. In addition, distinct benefits of the DASH diet among subgroups and personalized dietary programs for the primary prevention of VTE need further validation in the future.

In our analyses, individuals identified as high genetic risk via PRS exhibited similar incidence rates to FV Leiden or prothrombin G20210A heterozygotes but encompassed a population 150 times larger. Focusing solely on common thrombophilic mutations limits clinical and public health impact due to their small genetic explanation variance of thrombosis, while polygenic backgrounds can modulate these genetic risk factors. Given that current guidelines recommend thromboprophylaxis in the presence of FV Leiden and G20210A polymorphism, especially in homozygous or compound heterozygous states [55,56], promoting the use of PRS to improve the identification of high- and low-risk individuals is more clinically significant. Research has proved the limited motivational impact of genetic testing on lifestyle improvement, and some individuals may deteriorate their habits upon receiving genetic risk information [57]. This observation may potentially be due to a deterministic view of unchangeable genetic risk [58]. However, the compelling evidence of gene-diet interactions presented in our study challenges this deterministic view and could empower individuals to adopt optimized dietary plans for VTE prevention.

To our knowledge, the present study is the first to assess the possible additive interaction between genetic risk and dietary patterns on VTE. The results revealed that high genetic risk and a low-adherent DASH diet synergistically increased the risk of VTE, indicating that the excess risks of gene-related VTE may partly result from poor dietary exposure. It could be speculated that the DASH diet modifies the impact of genetic susceptibility on VTE. In the sex-specific analyses, higher DASH scores were associated with lower risks of VTE in both males and females. It should be noted that 14% of additional cases were ascribed to an interaction between low DASH scores and high genetic risk in males. Therefore, for individuals at high genetic risk, particularly males, adopting a DASH diet can lead to additional benefits in VTE prevention.

Unlike prior studies focusing on pharmacological prevention [[59], [60], [61]], our study uniquely identified a protective effect of the DASH diet on PE risk, offering a novel, nonpharmacological perspective on primary prevention. Evidence from randomized controlled trials indicates that the diet lowers proinflammatory protein levels, likely reducing vascular inflammation, metabolic stress, and clot formation [62]. Improvements in blood pressure, insulin sensitivity, and obesity, which were pivotal thrombosis risk factors, may further mediate its protective effects [22]. Additionally, the natriuretic effects and interaction with the renin-angiotensin-aldosterone system contribute to blood pressure regulation [22,63]. Nutrients such as alpha-linolenic acid, polyphenols, and omega-3 fatty acids, found in foods like nuts, olive oil, and oily fish, rapidly reduce inflammation and thrombosis, enhancing blood flow and preventing embolization [62]. Future mechanistic studies are needed to clarify biological pathways using biomarkers and hemodynamic data.

Our study still has limitations, including the use of a short baseline dietary intake measurement, the potential self-reported misclassification, and inability to capture longitudinal changes in dietary habits in the long term, though it was believed to be reasonably ranked food and beverage intake by the Food Frequency Questionnaire [64]. We attempted to mitigate dietary changes by excluding cancer patients. Observational nature also presented challenges in capturing causal relationships. It should be acknowledged that residual confounding cannot be fully ruled out due to the observational nature of the study. However, E-values, the minimum strength of an association that an unmeasured confounding would need to have to negate the observed effects, were estimated as sufficiently large to ensure the robustness of the conclusions drawn from the study. It should also be noted that using nonvalidated diagnosis codes from registries may introduce misclassification, which is likely nondifferential and could potentially lead to an underestimation of the observed associations. Additionally, the study population was limited to White European descent, limiting direct application to other ethnic groups. Despite efforts to mitigate these limitations, further investigations are advocated for in more diverse populations worldwide.

## 5 Conclusions

This large prospective cohort study revealed that the risk of incident VTE increased with higher PRS levels while decreasing with higher DASH scores. A low-adherent DASH diet combined with high genetic risk resulted in the highest risk of VTE. A high-adherent DASH diet reduced VTE risk across all genetic profiles.
