# Supplementary material for: Association between genetic risk and adherence to the Dietary Approaches to Stop Hypertension diet for developing venous thromboembolism
Source: Res Pract Thromb Haemost. 2025 Mar 12;9(2):102731. doi: 10.1016/j.rpth.2025.102731 (PMC11999685; doi:10.1016/j.rpth.2025.102731)
Supplement: Supplemental Material [file mmc1.docx]

## Supplementary methods

***Assessment of Covariates.*** The covariates, which could confuse the effect of dietary intake on VTE potentially, were selected based on prior knowledge and previous studies. ^1-4^ Potential confounding factors for VTE included age, gender, BMI, ethnicity, Townsend deprivation index, smoking status, drinking status, Metabolic Equivalent Task levels, systolic blood pressure (SBP), low–density lipoprotein (LDL), aspirin medication and history of medical conditions during the baseline assessment. BMI was calculated as the weight in kilograms divided by the square of the height in meters and further divided into four categories (18.5–24.9, < 18.5, 25–29.9, or ≥ 30 kg/m^2^). The Townsend deprivation index measures material deprivation using census variables on unemployment, overcrowding, car ownership, and home ownership.^5^ Smoking status was categorized as current, previous, or never smokers. Drinking status was assessed through the question “About how often do you drink alcohol?” and categorized as never, moderate (3 or 4 times a week, once or twice a week, 1–3 times a month, special occasions only), and heavy (daily or almost daily). According to the International Physical Activity Questionnaire,^6^ physical activity was assessed using the metabolic equivalent minutes per week, and participants were categorized into low, moderate, and high–intensity groups based on their reported time and exercise intensity. Moreover, cardiovascular disease (CVD), diabetes, hypertension, and lipidaemias might confound the relationship between the DASH diet and VTE.^7^ Therefore, these medical histories (yes or no) at the baseline were also separately taken into account eliminate the potential impact on the results. Covariates in PRS model additionally included the genotyping batches and the top eigenvectors for population substructure. For missing data imputations, we applied a missing indicator category for categorical variables and a median value for continuous variables.

***Subgroup and sensitivity analyses.*** In subgroup analyses, the models were re-run stratified by age, gender, Ethnicity, body mass index, smoking status, drinking status, physical activity and previous CVD/ hypertension/ lipidaemias/ diabetes to assess potential effect modification. We also performed several sensitivity analyses to enhance the robustness of our findings by, 1) restricting participants with complete covariates, 2) excluding participants with less than one year and two years of follow-up, respectively, 3) excluding participants who were diagnosed with VTE within the first year of follow-up, 4) restricting European British with complete covariates and imputation covariates, respectively, 5) excluding participants with extreme DASH-style food components. In addition, to minimize the likelihood of reverse causality, we first analyzed using Cox models additionally adjusted for prevalent diet-related illnesses at baseline. The E-value was calculated using the R package "EValue" and was designed to assess the potential effect of unmeasured confounders on the relationship between observed exposure and outcome. It represents how strong unmeasured confounding needs to be to fully account for the observed association. The most common genetic polymorphisms remain elusive for many clinicians and patients. Among them, the common genetic polymorphisms FV Leiden (rs6025:C>T) and prothrombin G20210A (rs1799963:G>A) are of particular interest. After excluding 71,086 subjects (17.3%) with missing genotype data, our final analysis included 9,012 VTE cases and 331,441 controls. Additionally, we identified 821 baseline subjects with anticoagulant deficiencies by mapping the ICD-10 code D68. To explore the effect of high DASH scores in preventing different VTE types based on the location of thrombosis, primary VTE cases were further classified into three subcategories: DVT alone, PE alone, and a combination of PE with DVT.

**Table S1. Food items and scoring for the 7 food groups in DASH.**

| **Food groups** | **Components** | **Field IDs** | **Total consumption^a^** | **Criteria for minimum score of 1** | **Criteria for maximum score of 5** |
| --- | --- | --- | --- | --- | --- |
| **Healthy food groups** | Vegetables | 1289-Cooked vegetable intake  1299-Salad/raw vegetable intake | 2 × Cooked + Salad/raw | Lowest quintile | Highest quintile |
|  | Fruits | 1309-Fresh fruit intake  1319-Dried fruit intake | 2 × Dried + Fresh |  |  |
|  | Low-fat dairy | 1408-Cheese intake  1418-Milk intake | Cheese + Milk |  |  |
|  | Whole grains | 1438-Bread intake  1458-Cereal intake | Bread + Cereal |  |  |
| **Unhealthy food groups^b^** | Red and processed meats | 1349-Processed meat intake  1369-Beef intake  1379-Lamb/mutton intake  1389-Pork intake | Processed meat + Beef + Lamb + Pork | Highest quintile | Lowest quintile |
|  | Sodium | 1478-Salt added to food | Frequency of adding salt to food |  |  |
|  | Sugar-sweetened beverages | 6144-Never consumes drinks containing sugar | Eat any of the foods contains sugar |  |  |

**^a^** Total consumptions for vegetables and fruits vary based on their types, recommended in DASH diet plan.

^b^Higher quintiles represent higher intake. In constructing the DASH score, higher intake (higher quintiles) of these components received lower scores.

**Table S2. Comparisons of baseline characteristics between the non-VTE and VTE participants.**

| **Characteristics,**  **n (%) or median (IQR)** | **Non-VTE  (N=400 996)** | **Incident VTE  (N=10 543)** |
| --- | --- | --- |
| Age (years), continuous | 57.0 (49.0, 63.0) | 61.0 (55.0, 65.0) |
| Age (years), categories |  |  |
| <60 | 237 783 (59.3) | 4 374 (41.5) |
| ≥60 | 163 213 (40.7) | 6 169 (58.5) |
| Gender |  |  |
| Female | 215 815 (53.8) | 5 042 (47.8) |
| Male | 185 181 (46.2) | 5 501 (52.2) |
| Ethnicity |  |  |
| Other | 68 945 (17.2) | 1 520 (14.4) |
| European British | 332 051 (82.8) | 9 023 (85.6) |
| BMI (kg/m^2^) |  |  |
| 18.5-24.9 | 132 890 (33.1) | 2364 (22.4) |
| <18.5 | 2026 (0.5) | 27 (0.3) |
| 25-29.9 | 170 608 (42.5) | 4 426 (42.0) |
| ≥30 | 93 636 (23.4) | 3 661 (34.7) |
| causality | 1 836 (0.5) | 65 (0.6) |
| Smoking status |  |  |
| Never | 221 966 (55.4) | 5 127 (48.6) |
| Previous | 136 874 (34.1) | 4 078 (38.7) |
| Current | 41 044 (10.2) | 1 299 (12.3) |
| Missing | 1112 (0.3) | 39 (0.4) |
| Drinking status |  |  |
| Non-drinkers | 72 501 (18.1) | 2 328 (22.1) |
| Moderate | 244 712 (61.0) | 6 008 (57.0) |
| Heavy | 83 609 (20.9) | 2 197 (20.8) |
| Missing | 174 (0.0) | 10 (0.1) |
| Physical activity |  |  |
| Low | 62 017 (15.5) | 1 715 (16.3) |
| Moderate | 136 198 (34.0) | 3 426 (32.5) |
| High | 134 897 (33.6) | 3 416 (32.4) |
| Missing | 67 884 (16.9) | 1 986 (18.8) |
| SBP (mm Hg) | 136.0 (124.5, 149.0) | 138.5 (127.0, 151.0) |
| DBP (mm Hg) | 82.0 (75.0, 89.0) | 83.0 (76.5, 89.5) |
| LDL (mmol/L) | 3.5 (3.0, 4.1) | 3.5 (2.9, 4.1) |
| Townsend index | -2.2 (-3.7, 0.4) | -2.1 (-3.6, 0.7) |
| Aspirin |  |  |
| Never | 344 126 (85.8) | 8 501 (80.6) |
| Previous | 53 100 (13.2) | 1 915 (18.2) |
| Missing | 3 770 (0.9) | 127 (1.2) |
| History of CVD |  |  |
| No | 377 031 (94.0) | 9 548 (90.6) |
| Yes | 23 965 (6.0) | 995 (9.4) |
| History of diabetes |  |  |
| No | 390 631 (97.4) | 10 094 (95.7) |
| Yes | 10 365 (2.6) | 449 (4.3) |
| History of hypertension |  |  |
| No | 298 828 (74.5) | 6 901 (65.5) |
| Yes | 102 168 (25.5) | 3 642 (34.5) |
| History of lipidaemias |  |  |
| No | 344 032 (85.8) | 8 531 (80.9) |
| Yes | 56 964 (14.2) | 2 012 (19.1) |

Abbreviations: VTE, venous thromboembolism; IQR, interquartile range; BMI, body mass index; SBP, systolic blood pressure; DBP, diastolic blood pressure; LDL, low-density lipoprotein; CVD, cardiovascular disease.

**Table S3. Associations of FV Leiden, G20210A polymorphism and coagulation defects with risk of incident VTE.**

| **Subgroups** | **No. of cases/ Total no.** | **Person-year** | **Incidence Rate ‰**  **(95% CI)** | **Model 1 HR (95% CI) ^a^** | **Model 2 HR**  **(95% CI) ^a^** | **Model 3 HR**  **(95% CI) ^a^** | **E-value**  **(CI) ^b^** |
| --- | --- | --- | --- | --- | --- | --- | --- |
| FV Leiden |  |  |  |  |  |  |  |
| C vs. T | 9 012/340 453 | 4 434 537 | 2.03 (1.99, 2.07) | **2.01 (1.87, 2.16)** | **2.03 (1.89, 2.18)** | **2.04 (1.89, 2.19)** | 3.49 (3.19) |
| CC | 8262/325 456 | 4 242 000 | 1.95 (1.91, 1.99) | 1.00 (ref.) | 1.00 (ref.) | 1.00 (ref.) |  |
| CT | 733/14 856 | 190 816 | 3.84 (3.57, 4.13) | **1.99 (1.84, 2.14)** | **2.00 (1.86, 2.16)** | **2.01 (1.86, 2.16)** | 3.43 (3.13) |
| TT | 17/141 | 1 720 | 9.88 (5.76, 15.82) | **5.53 (3.44, 8.90)** | **5.81 (3.61, 9.35)** | **5.84 (3.63, 9.40)** | 11.16 (6.72) |
| G20210A |  |  |  |  |  |  |  |
| G vs. A | 9 012/340 453 | 4 434 537 | 2.03 (1.99, 2.07) | **1.43 (1.30, 1.56)** | **1.43 (1.31, 1.57)** | **1.43 (1.31, 1.57)** | 2.22 (1.94) |
| GG | 8 530/327 328 | 4 264 361 | 2.00 (1.96, 2.04) | 1.00 (ref.) | 1.00 (ref.) | 1.00 (ref.) |  |
| GA | 475/13 041 | 169 113 | 2.81 (2.56, 3.07) | **1.41 (1.29, 1.55)** | **1.42 (1.29, 1.55)** | **1.42 (1.29, 1.55)** | 2.18 (1.90) |
| AA | 7/84 | 1 063 | 6.59 (2.65, 13.57) | **3.17 (1.51, 6.65)** | **3.37 (1.60, 7.06)** | **3.41 (1.62, 7.15)** | 6.27 (2.63) |
| Coagulation defects |  |  |  |  |  |  |  |
| No | 10 354/409 996 | 5 336 332 | 1.94 (1.90, 1.98) | 1.00 (ref.) | 1.00 (ref.) | 1.00 (ref.) |  |
| Yes | 151/821 | 8 667 | 17.42 (14.75, 20.43) | **8.54 (7.27, 10.02)** | **7.63 (6.50, 8.96)** | **7.53 (6.41, 8.85)** | 14.55 (12.30) |

Abbreviations: DASH, Dietary Approaches to Stop Hypertension; PRS, polygenic risk scores; VTE, venous thromboembolism; HR, hazard ratio; CI, confidence interval; SD, standard deviation; Q, quintile.

^a^ Model 1: age and gender; Model 2: Model 1 + BMI + smoking + drinking + Townsend deprivation index + physical activity + ethnicity; Model 3: Model 2 + SBP + LDL+ aspirin medication+ CAD history+ diabetes history + hypertension history + lipidaemias history. The boldface value denotes significant differences (*P* < 0.05).

^b^ The E-value was calculated based on the full-adjusted HR and lower bound of 95% CI.

**Table S4. Association of high DASH diet scores with incident VTE risk stratified by FV Leiden, G20210A, and coagulation Defects.**

| **Subgroups** | **No. of cases/ Total no.** | **Person-year** | **Incidence Rate ‰**  **(95% CI)** | **Adjusted HR (95% CI)^a^** | ***P* value** | **E value (CI)** | |
| --- | --- | --- | --- | --- | --- | --- | --- |
| FV Leiden |  |  |  |  |  |  |  |
| CC | 1 514/56 417 | 732 764 | 2.07 (1.96, 2.17) | **0.85 (0.79, 0.91)** | <0.001 | 1.64 (1.44) |  |
| CT | 133/2 629 | 33 636 | 3.95 (3.31, 4.69) | 0.89 (0.71, 1.12) | 0.318 | 1.50 (1.00) |  |
| G20210A |  |  |  |  |  |  |  |
| GG | 1 560/56 809 | 737 519 | 2.12 (2.01, 2.22) | **0.85 (0.80, 0.91)** | <0.001 | 1.63 (1.43) |  |
| GA | 88/2 239 | 28 899 | 3.05 (2.44, 3.75) | 0.91 (0.69, 1.21) | 0.519 | 1.43 (1.00) |  |
| Coagulation defects |  |  |  |  |  |  |  |
| No | 1 896/69 663 | 903691 | 2.10 (2.00, 2.19) | **0.87 (0.82, 0.93)** | <0.001 | 1.56 (1.37) |  |
| Yes | 9/121 | 1470 | 6.12 (2.8, 11.62) | 1.25 (0.51, 3.09) | 0.623 | 1.82 (1.00) |  |

^a^Models were adjusted for age, BMI, gender, ethnicity, Townsend deprivation index, smoking status, drinking status, MET, SBP, LDL, aspirin medication, history of CVD, history of diabetes, history of hypertension and history of lipidaemias. The value in bold denotes significance.

**Table S5. Associations of DASH diet score with risk of VTE Types.**

| **VTE type** | **No. of cases/ Total no.** | **Person-year** | **Incidence Rate ‰**  **(95% CI)** | **Adjusted HR (95% CI)^a^** | ***P* value** | **E value (CI)** |  |
| --- | --- | --- | --- | --- | --- | --- | --- |
| DVT alone |  |  |  |  |  |  | |
| Q1 | 1 162/100 708 (1.15) | 1 318 292 | 0.88 (0.83, 0.93) | 1.00 (ref.) |  |  | |
| Q5 | 905/68 784 (1.32) | 897 488 | 1.01 (0.94, 1.08) | 0.95 (0.87, 1.04) | 0.279 | 1.28 (1.00) | |
| PE alone |  |  |  |  |  |  | |
| Q1 | 1 183/100 729 (1.17) | 1319 758 | 0.90 (0.85, 0.95) | 1.00 (ref.) |  |  | |
| Q5 | 796/68 675 (1.16) | 898 164 | 0.89 (0.83, 0.95) | **0.80 (0.73, 0.88)** | <0.001 | 1.80 (1.53) | |
| PE DVT |  |  |  |  |  |  | |
| Q1 | 243/99 789 (0.24) | 1312002 | 0.19 (0.16, 0.21) | 1.00 (ref.) |  |  | |
| Q5 | 204/68 083 (0.30) | 893057 | 0.23 (0.2, 0.26) | 1.01 (0.84, 1.22) | 0.912 | 1.12 (1.00) | |

^a^Models were adjusted for age, BMI, gender, ethnicity, Townsend deprivation index, smoking status, drinking status, MET, SBP, LDL, aspirin medication, history of CVD, history of diabetes, history of hypertension and history of lipidaemias. The value in bold denotes significance.

**Table S6. Sensitivity analyses of DASH diet score on VTE.**

| Sensitive analyses | **Adjusted HR (95% CI)^a^** | ***P* value** | **E value (CI)** | |
| --- | --- | --- | --- | --- |
| Complete covariates  (N=501 098) | **0.87 (0.81, 0.94)** | <0.001 | 1.55 (1.32) |  |
| European British with imputed covariates  (N=408 722) | **0.85 (0.80, 0.91)** | <0.001 | 1.62 (1.43) |  |
| European British with complete covariates  (N=341 074) | **0.85 (0.79, 0.92)** | <0.001 | 1.62 (1.38) |  |
| Excluded one-year follow-up  (N=410 547) | **0.86 (0.81, 0.92)** | <0.001 | 1.59 (1.40) |  |
| Excluded two-year follow-up  (N=409 207) | **0.86 (0.81, 0.92)** | <0.001 | 1.60 (1.41) |  |
| Excluded VTE occurrence within one-year follow-up  (N=411 008) | **0.86 (0.81, 0.92)** | <0.001 | 1.58 (1.40) |  |
| Excluded food components above the 99th percent  (N=411 539) | **0.87 (0.82, 0.92)** | <0.001 | 1.57 (1.39) |  |

^a^Models were adjusted for age, BMI, gender, ethnicity, Townsend deprivation index, smoking status, drinking status, MET, SBP, LDL, aspirin medication, history of CVD, history of diabetes, history of hypertension and history of lipidaemias. The value in bold denotes significance.

**Table S7. Additive and multiplicative interactions between DASH diet score and PRS on risk of incident VTE.**

| Participants | **Medium genetic risk^a^** | | |  | **High genetic risk^a^** | | | ***P* for INT_M_** |
| --- | --- | --- | --- | --- | --- | --- | --- | --- |
|  | **RERI (95% CI)** | **AP (95% CI)** | **S (95% CI)** |  | **RERI (95% CI)** | **AP (95% CI)** | **S (95% CI)** |  |
| **Total** | 0.02 (-0.21, 0.25) | 0.01 (-0.13, 0.15) | 1.03 (0.71, 1.50) |  | **0.29 (0.03, 0.55)** | **0.11 (0.01, 0.20)** | **1.20 (1.00, 1.43)** | 0.793 |
| **Male** | 0.13 (-0.20, 0.46) | 0.08 (-0.12, 0.28) | 1.24 (0.66, 2.32) |  | **0.46 (0.08, 0.85)** | **0.16 (0.03, 0.29)** | **1.32 (1.01, 1.72)** | 0.482 |
| **Female** | -0.30 (-0.71, 0.10) | -0.19 (-0.44, 0.06) | 0.67 (0.43, 1.06) |  | 0.11 (-0.33, 0.54) | 0.04 (-0.12, 0.20) | 1.07 (0.81, 1.41) | 0.604 |

Abbreviations: DASH, Dietary Approaches to Stop Hypertension; PRS, polygenic risk score; VTE, venous thromboembolism; HR, hazard ratio; CI, confidence interval; RERI: relative excess risk due to interaction; AP, attributable proportion; S, synergy index; INT_M_, multiplicative interaction.

**^a^**Models were adjusted for age, BMI, gender, ethnicity, Townsend deprivation index, smoking status, drinking status, MET, SBP, LDL, aspirin medication, history of CAD, history of diabetes, history of hypertension, history of lipidaemias, genotyping batch, and the first five genetic principal components. The value in bold denotes significance of an additive interaction.


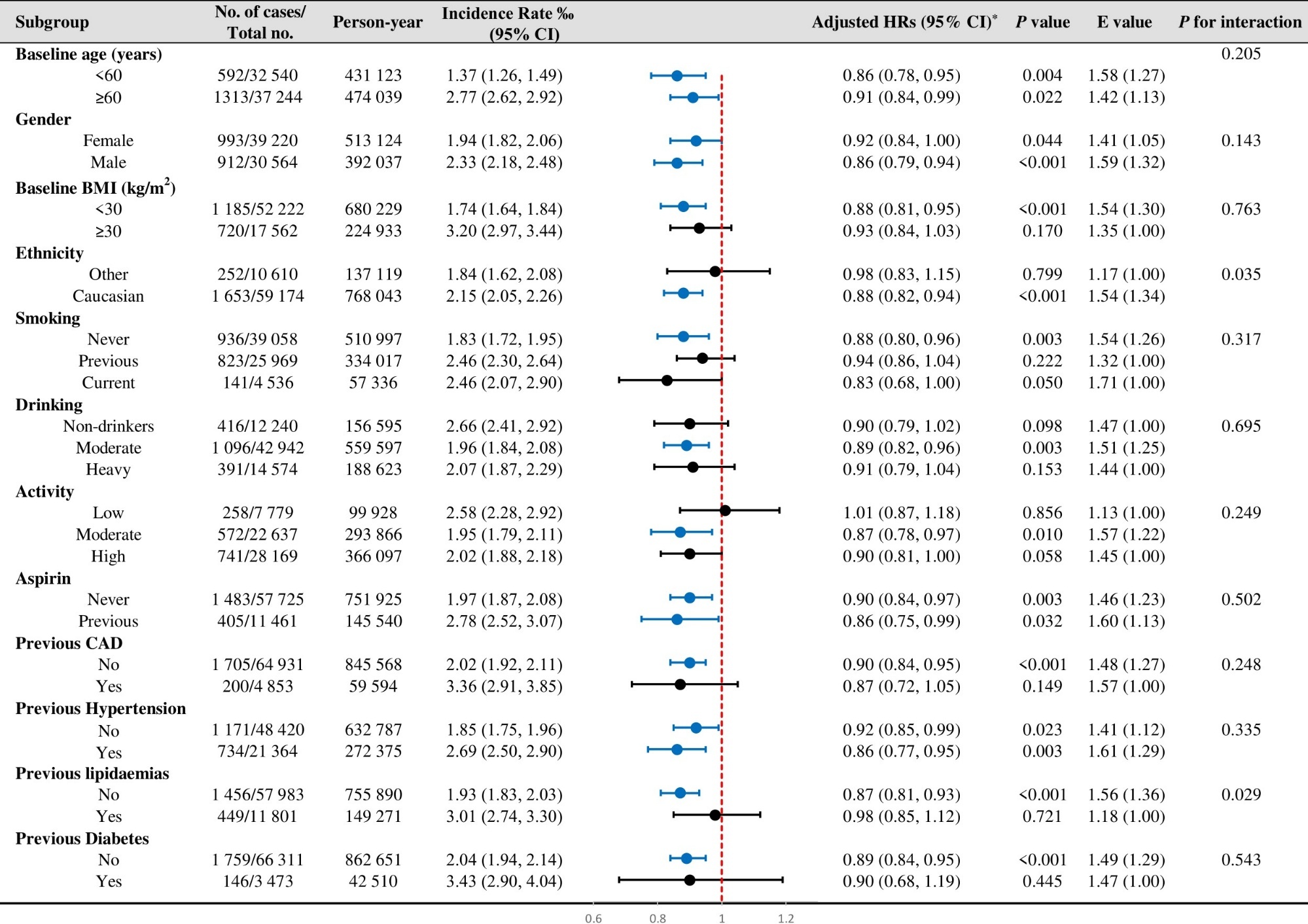
**Figure S1. Multivariate HRs of associations of DASH diet scores with incident VTE across different subgroups.**

**^a^**Models were adjusted for age, BMI, gender, ethnicity, Townsend deprivation index, smoking status, drinking status, MET, SBP, LDL, aspirin medication, history of CVD, history of diabetes, history of hypertension and history of lipidaemias.


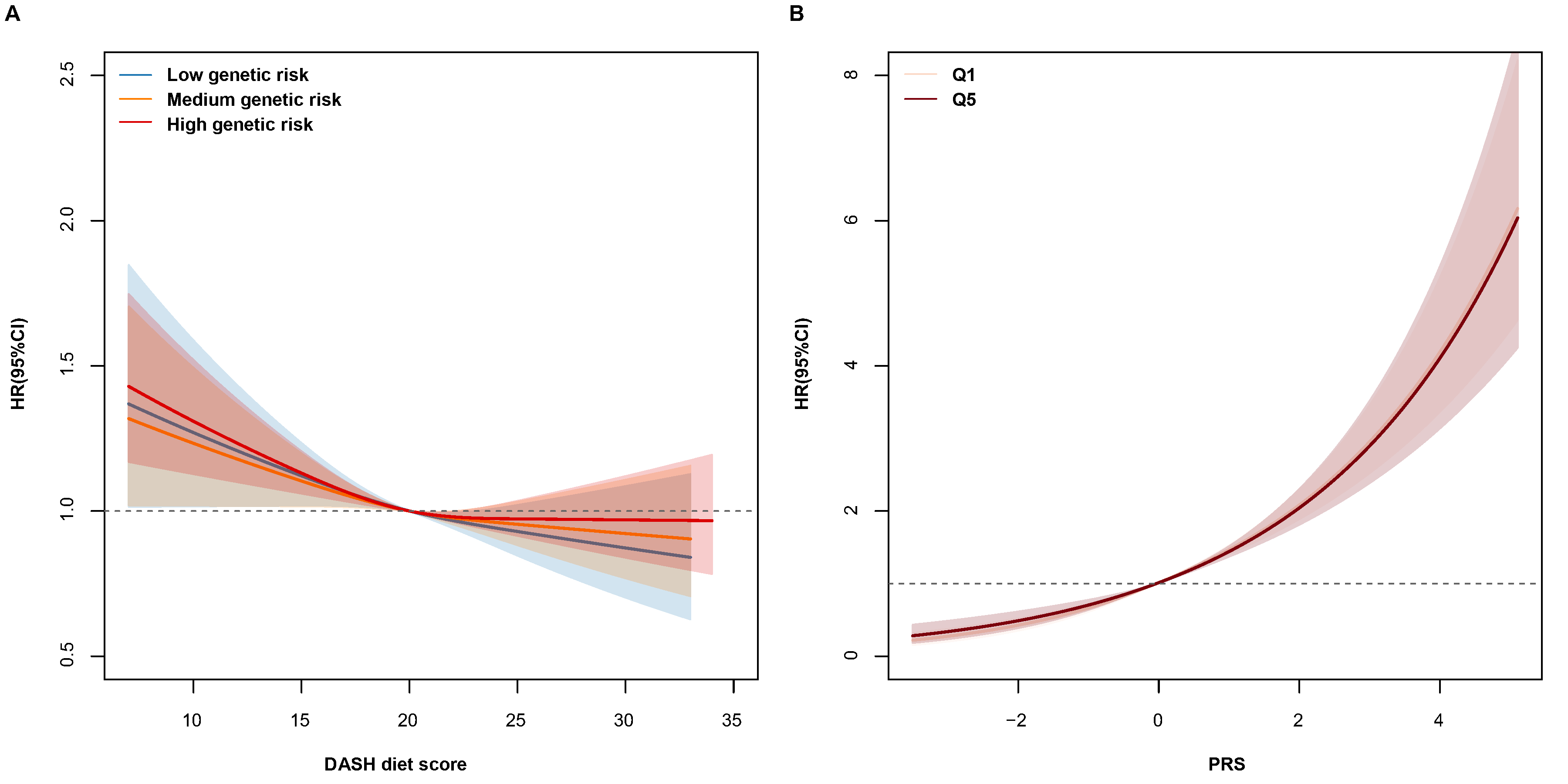
**Figure S2. Exposure–response associations between DASH diet score (A) and PRS (B) with risk of incident VTE.** All HRs were adjusted for age, BMI, gender, ethnicity, Townsend deprivation index, smoking status, drinking use, physical activity, SBP, LDL, aspirin medication, genotyping batch, and the first five genetic principal components.

Abbreviations: DASH, Dietary Approaches to Stop Hypertension; Q, quintile; PRS, polygenic risk score; HR, hazard ratio; CI, confidence interval.


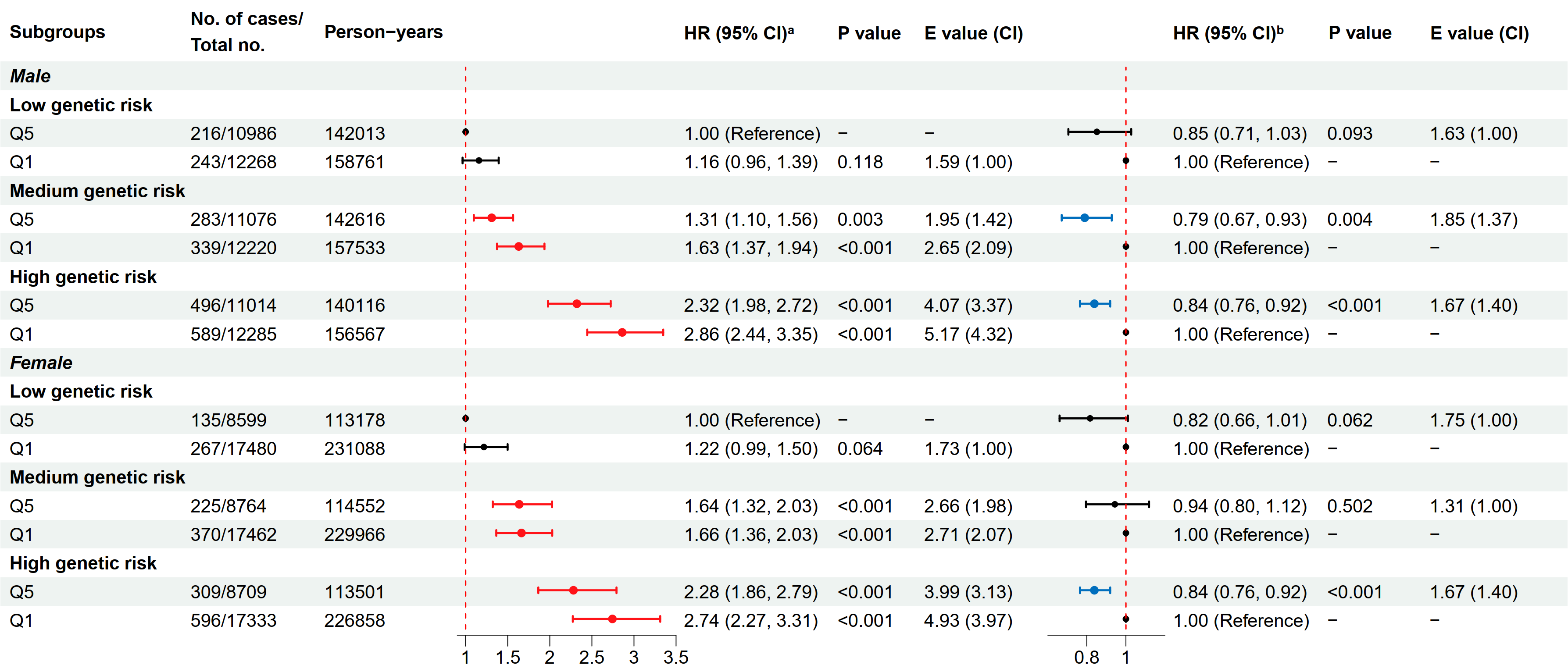
 **Figure S3. Joint effects and interaction of DASH diet score and genetic risk stratified by gender.** All HRs were adjusted for age, BMI, sex, ethnicity, Townsend deprivation index, smoking status, drinking use, physical activity, SBP, LDL, aspirin medication, history of CVD, diabetes, hypertension or lipidaemias, genotyping batch, and the first five genetic principal components.

^a^ Participants with the lowest quintile of DASH score and high-genetic risk were set as the reference.

^b^ Participants with the lowest quintile of DASH score in each genetic risk stratification were set as the reference, respectively.

Abbreviations: DASH, Dietary Approaches to Stop Hypertension; Q, quintile; PRS, polygenic risk score; HR, hazard ratio; CI, confidence interval.


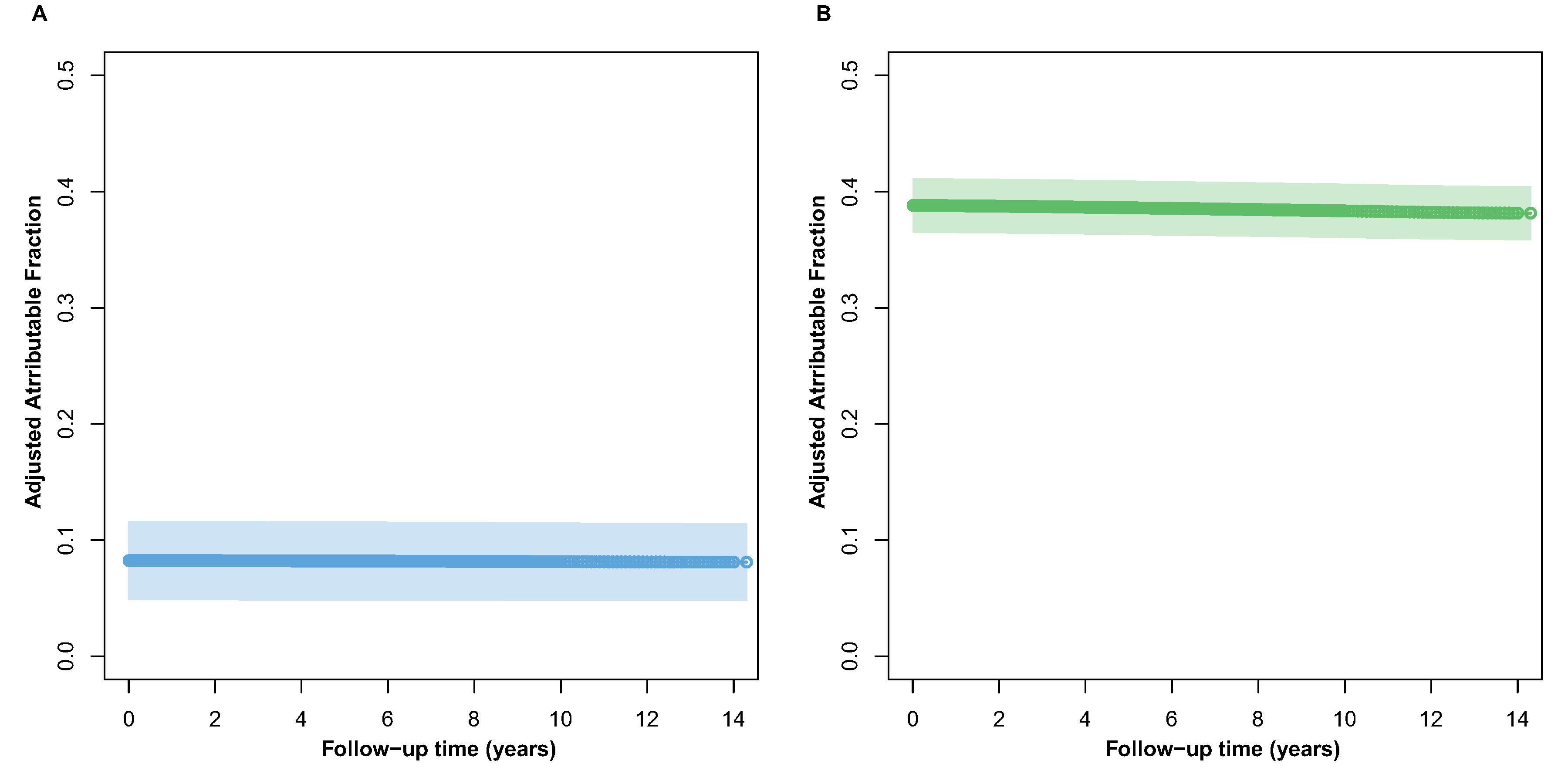
**F****igure S4. The adjusted attributable fraction of DASH diet score (A) and PRS (B) for incident VTE.**

The dots in lines are attributable fractions adjusted for age, gender, BMI, Townsend deprivation index, smoking status, drinking status, physical activity, aspirin medication, SBP, LDL, Ethnicity, and history of CVD, diabetes, hypertension or lipidaemias (adjusted attributable fractions of PRS are further adjusted by genotyping batch and the first five principal components of ancestry), indicates the proportion of the VTE that would be eliminated if participants were not in the lowest quintile of the DASH diet score (A) or highest tertile of PRS (B) during the follow-up time.

## Reference

1. Fung TT, Chiuve SE, McCullough ML, Rexrode KM, Logroscino G, Hu FB. Adherence to a DASH-style diet and risk of coronary heart disease and stroke in women. *Arch Intern Med*. Apr 14 2008;168(7):713-20. doi:10.1001/archinte.168.7.713

2. Fitzgerald KC, Chiuve SE, Buring JE, Ridker PM, Glynn RJ. Comparison of associations of adherence to a Dietary Approaches to Stop Hypertension (DASH)-style diet with risks of cardiovascular disease and venous thromboembolism. *J Thromb Haemost*. Feb 2012;10(2):189-98. doi:10.1111/j.1538-7836.2011.04588.x

3. Filippou CD, Tsioufis CP, Thomopoulos CG, et al. Dietary Approaches to Stop Hypertension (DASH) Diet and Blood Pressure Reduction in Adults with and without Hypertension: A Systematic Review and Meta-Analysis of Randomized Controlled Trials. *Advances in nutrition (Bethesda, Md)*. Sep 1 2020;11(5):1150-1160. doi:10.1093/advances/nmaa041

4. Yuan S, Bruzelius M, Hakansson N, Akesson A, Larsson SC. Lifestyle factors and venous thromboembolism in two cohort studies. *Thromb Res*. Jun 2021;202:119-124. doi:10.1016/j.thromres.2021.03.024

5. Adams J, Ryan V, White M. How accurate are Townsend Deprivation Scores as predictors of self-reported health? A comparison with individual level data. *J Public Health (Oxf)*. Mar 2005;27(1):101-6. doi:10.1093/pubmed/fdh193

6. Craig CL, Marshall AL, Sjostrom M, et al. International physical activity questionnaire: 12-country reliability and validity. *Med Sci Sports Exerc*. Aug 2003;35(8):1381-95. doi:10.1249/01.MSS.0000078924.61453.FB

7. Mahmoodi BK, Cushman M, Anne Naess I, et al. Association of Traditional Cardiovascular Risk Factors With Venous Thromboembolism: An Individual Participant Data Meta-Analysis of Prospective Studies. *Circulation*. Jan 3 2017;135(1):7-16. doi:10.1161/CIRCULATIONAHA.116.024507

8. VanderWeele TJ, Ding P. Sensitivity Analysis in Observational Research: Introducing the E-Value. *Ann Intern Med*. Aug 15 2017;167(4):268-274. doi:10.7326/M16-2607
